# Flare up of choroiditis and choroidal neovasculazation associated with punctate inner choroidopathy during early pregnancy

**DOI:** 10.4103/0301-4738.77039

**Published:** 2011

**Authors:** Vinita G Rao, Girish S Rao, Nilesh S Narkhede

**Affiliations:** Uveitis Services, and Shri Ganapati Netralaya, Jalna, Maharashtra, India; 1 Vitreo-Retinal Services, Shri Ganapati Netralaya, Jalna, Maharashtra, India

**Keywords:** Choroidal neovascular membrane, lucentis, photodynamic therapy, pregnancy, punctate inner choroidopathy

## Abstract

A 28-year-old, healthy female, who had a recent repeated history of miscarriage, presented with bilateral choroidal neovascular membranes (CNVM), for which she received photodynamic therapy with three doses of lucentis, at intervals of one month each, to which she responded. After five months, the patient again presented with complaints of diminution of vision since 15 days. She had a history of miscarriage two days before presenting to our clinic. CNVM was scarred at this time and the fundus picture showed multiple small punctate spots around the fovea at the level of the choroid, which showed early hyperfluroscence on fundus fluorescein angiography, suggestive of punctate inner choroidopathy. She was advised systemic steroids, to which she responded dramatically.

Punctate inner choroidopathy (PIC), first described by Watzke in 1984, is one of the white dot syndromes for which the exact etiology is not known.[1] It is an idiopathic inflammatory disorder of the choroid that typically affects myopic women of 13 – 45 years.[2] It is a bilateral disease, which may be asymmetric.[2] Patients present with complaints of flashes of light, floaters, scotoma or decrease in central visual acuity.[1] The anterior chamber and vitreous is usually quiet. Small (100 – 300 µ) punctate, yellowish-white lesions, confined to the inner choroids and retinal pigment epithelium are seen at the posterior pole.[2] Fundus fluorescein angiography (FFA) shows early hyperfluroscent dots that stain in the late pictures. With time, the lesions evolve and coalesce into atrophic scars that later on become pigmented. Most of the patients have an excellent prognosis with spontaneous recovery over a period of four to six weeks.[2] About 10 – 40% of the patients present with choroidal neovascular membrane (CNVM) as a late complication, which is a cause for decrease in visual acuity.[2] Usually non-infectious uveitis entities such as idiopathic punctate inner choroidopathy (PIC) are known to be suppressed during pregnancy and aggravate in the postpartum period.[3] We report a case of PIC with CNVM developing during pregnancy and the exacerbation of choroiditis in the first trimester.

## Case Report

**Figure 1:**
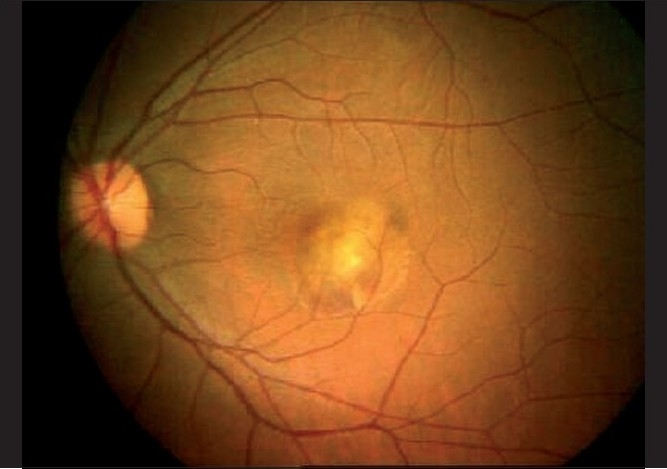
(Flare up of PIC in pregnancy) Fundus photograph of the first visit showing active CNVM (left eye) without any signs of choroiditis

**Figure 2:**
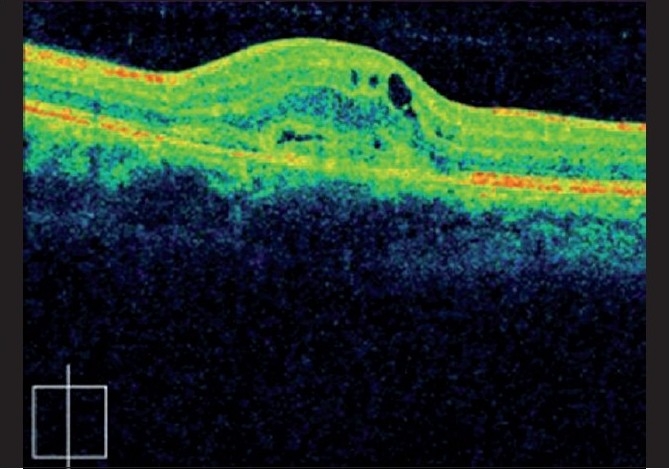
(Flare up of PIC in pregnancy) OCT showing Type2 CNVM, (i.e., between RPE and neurosensory retina) characteristic of PIC, along with fluid collection in the adjacent neurosensory retina

**Figure 3:**
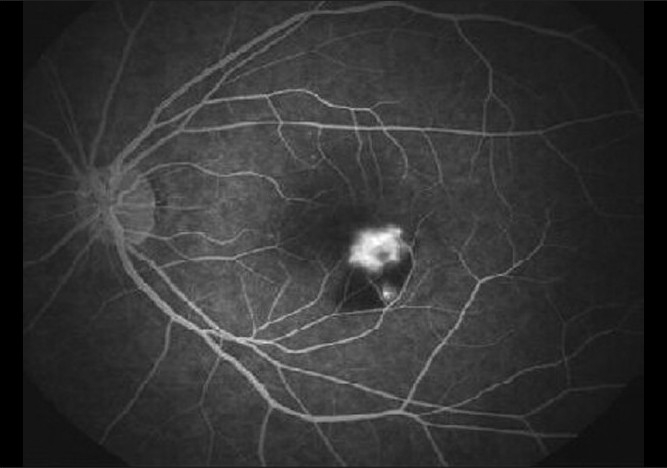
(Flare up of PIC in pregnancy) FFA revealed active leakage from the CNVM as shown by increase in fluorescence from the choroidal neovascularization

**Figure 4:**
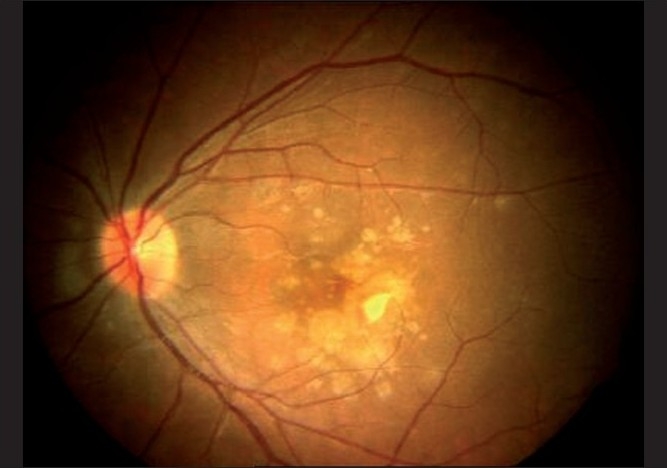
(Flare up of PIC in pregnancy) Fundus photograph showing multiple yellowish punctate lesions on the posterior pole at the level of the choroid and RPE with the scarred CNVM

**Figure 5:**
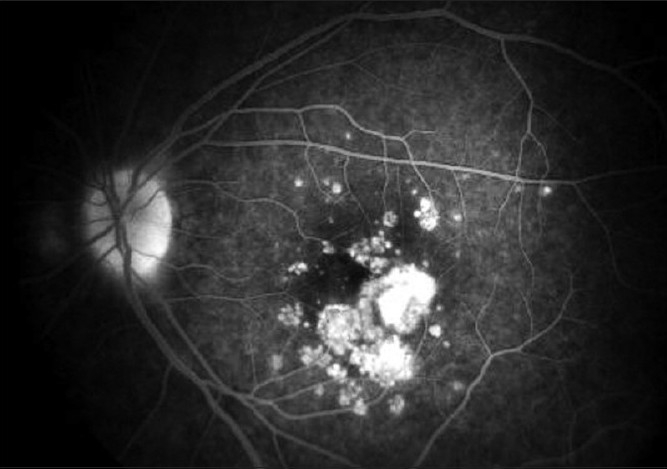
(Flare up of PIC in pregnancy) FFA revealing hyperfluroscence spots corresponding to the clinical yellowish lesions. Note, the CNVM was inactive at this time as confirmed by the FFA

**Figure 6:**
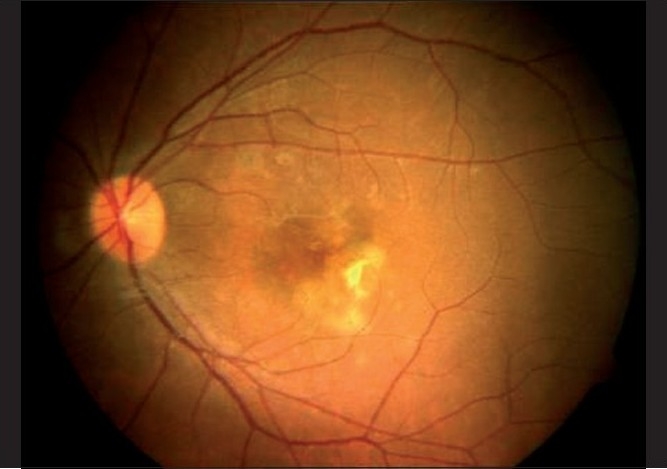
(Flare up of PIC in pregnancy) Fundus photograph revealing healed choroiditis after a course of systemic steroids

A 28-year-old, healthy female patient, who had a recent repeated history of miscarriage, presented with complaints of blurring of vision in both eyes. There was no antecedent history of viral illness. Her best corrected visual acuity was 20 / 400 (< N36) and 20 / 50 (N6) in the right and left eyes, respectively. She was a myope with -2.75 diopter (D) and -3.50D in the right and left eyes, respectively. On examination, the anterior chamber and vitreous were quiet. Fundus examination of the right eye showed a scarred CNVM, while that of the left eye showed an active CNVM [Fig. 1]. Optical coherence tomography (OCT) of the left eye showed the presence of type II CNVM (i.e., between the retinal pigment epithelium and neurosensory retina, Fig. 2), which was confirmed to be active on FFA, with a branched out configuration [Fig. 3]. The patient received PDT with three doses of lucentis at intervals of one month each and the visual acuity in the left eye improved from 20 / 50 to 20 / 30. indirectly indicating no signs of choroiditis. Five months later, the patient came with complaints of diminution of vision again. During this period she had been under bed rest for a threatened abortion. When she presented to us, she had a history of miscarriage two days back and had complaints of diminution of vision for two weeks. Her visual acuity in the left eye was 20 / 50, with the fundus revealing multiple yellowish lesions about 50 – 100 µ, at the level of the choroids around the scarred CNVM [Fig. 4]. FFA of the left eye showed scarred CNVM, with no leakage and small punctuate early hyperfluroscent spots corresponding to clinical lesions, with late leakage [Fig. 5]. On the basis of the present clinical features and the FFA picture, she was now diagnosed with PIC and was advised intravenous (IV) methyl prednisolone 1 gm/day for three days followed by oral steroids (Prednisolone 1 mg/kg/day) in tapering doses. Her visual acuity had improved to 20 / 30 (N6) at the one-week follow-up, with resolving choroiditis [Fig. 6].

## Discussion

Punctate inner choroidopathy is an idiopathic, inflammatory, multifocal choroidopathy of unknown etiology. Most of the patients with PIC have excellent prognosis, with spontaneous recovery.[1] CNVM is a known complication. In our patient CNVM had already scarred in one eye and was active in the other eye when she presented to us. As most of the cases of PIC resolve spontaneously, we presumed that previous attacks may have been unnoticed. Her pregnancy had been eventful with a stillbirth, and hence, we believed that the decrease in vision in the right eye also went unnoticed. The CNVM in our patient is typical of that seen in PIC. The FFA showed the typical branched out configuration of the CNVM, which is characteristic of PIC, as it is formed by numerous small punctuate lesions.[2]

Our patient had developed CNVM for the first time during pregnancy. Although there are no reports of CNVM developing for the first time in PIC during pregnancy, recurrences in pregnancy have been reported. This may be due to hormonal and hemodynamic changes. Excess levels of corticosteroids alter retinal pigment epithelium and choriocapillary permeability. Also, the surplus level of the vascular endothelial growth factor (VEGF) and placental growth factor (PGF), which control the growth of the placenta, also help in the growth of CNVM.[4,5] The physiological changes during pregnancy such as increase in blood volume and cardiac output may exacerbate choroidal vessels and retinal pigment epithelium damage.[4]

As with other non-infectious uveitic conditions, the activity of PIC is also suppressed during pregnancy and exacerbated in the postpartum period.[3] However, in our patient there was a flare up of choroiditis in the first trimester. There are few reports showing flare up of choroiditis during early pregnancy, which resolves as the duration of pregnancy increases.[3,6,7] The mechanism by which pregnancy influences the activity of autoimmune diseases is not understood completely. The progressive increase in serum cortisol level during pregnancy and decrease in the postpartum period might be an attractive explanation.[3] This could also be explained by the fact that normal pregnancy is a state associated with a shift from T helper cell type1 (TH1) cell-mediated immunity toward T helper cell type2 (TH2) humoral immunity. Maternal TH1 is proinflammatory, while TH2 secreted by the fetoplacental unit inhibits the TH1 response. This overall TH2 / TH1 bias during pregnancy not only explains the tolerance of the fetus by the mother, but also amelioration of autoimmune diseases during pregnancy. The postpartum reversal to the TH1 / TH2 bias could explain the postpartum flare up of autoimmune diseases.[3]

Therefore, we presume that the flare up of PIC, which was noticed by our patient would probably have subsided had her pregnancy continued, although it worsened due to the miscarriage.

Treatment of the choroiditis and CNVM may pose a problem in such patients, especially in an already eventful pregnancy or in a precious pregnancy. Our patient already had a miscarriage; and then we treated her with systemic steroids.[8] However, in case systemic steroids were contraindicated in future pregnancies, we might need to consider intravitreal triamcinolone.

This report highlights the fact that CNVM may be the first sign of PIC and the possibility of worsening of choroiditis or CNVM during pregnancy must be explained to the patients.
